# Tonsillar Actinomycosis Simulating an Oropharyngeal Neoplasm in a 67-Year-Old Man With Diabetes Mellitus, Hypertension, and Ischemic Heart Disease: A Case Report From King Faisal General Hospital, Hofuf, Saudi Arabia

**DOI:** 10.7759/cureus.113480

**Published:** 2026-07-27

**Authors:** Oluwarotimi O Bamiro, Babatope L Awosusi, Hussain A AlSaleh, Eman H Essa Alkhalaf

**Affiliations:** 1 Histopathology, King Faisal General Hospital, Hofuf, SAU; 2 Histopathology, King Khalid Hospital, Al Majmaah, SAU; 3 Otolaryngology, King Faisal General Hospital, Hofuf, SAU; 4 Haematology, King Faisal General Hospital, Hofuf, SAU

**Keywords:** actinomycosis mimicking malignancy, elderly patient, histopathology examination, oropharyngeal neoplasm, tonsillar actinomycosis

## Abstract

Actinomyces species constitute part of the normal microbial flora of the oral cavity. When mucosal integrity is disrupted, these organisms can produce invasive cervicofacial infections. Tonsillar actinomycosis can closely mimic an aggressive oropharyngeal malignancy in elderly patients with significant comorbidities.

A 67-year-old man with type 2 diabetes mellitus, hypertension, and ischemic heart disease presented with a chronic progressively enlarging right tonsillar mass accompanied by throat discomfort. Clinical assessment and computerized tomography imaging scan revealed a 42 mm × 24 mm × 47 mm avidly enhancing, lobulated, exophytic lesion centered in the right tonsillar fossa with apparent extension toward the tongue base and pterygoid musculature, raising strong suspicion for malignancy. Surgical excision demonstrated a well-circumscribed, non-infiltrative mass with an intact capsule. Histologic examination showed filamentous bacterial colonies and sulfur granules typical of actinomycosis on hematoxylin and eosin, PAS (periodic acid-Schiff), and GMS (Grocott methenamine silver stains). An extensive immunohistochemical panel (cytokeratin, CD (cluster of differentiation) 3, CD20, CD45, desmin, and p16) was negative, effectively excluding squamous cell carcinoma, lymphoma, and mesenchymal neoplasms. The patient received postoperative antibiotics and analgesics; symptoms resolved promptly, and he remained disease-free on follow-up.

Early surgical removal combined with careful histopathologic evaluation (including special stains and immunohistochemistry) and extended penicillin-based antimicrobial therapy permits accurate diagnosis, avoids unnecessary radical oncologic procedures, and yields excellent outcomes.

## Introduction

Bacteria belonging to the genus Actinomyces are anaerobic, filamentous, Gram-positive organisms that normally colonize the oral cavity, gastrointestinal tract, and female genital tract as commensals. Cervicofacial actinomycosis accounts for roughly half to three-fifths of all human infections caused by these organisms [1]. Isolated involvement of the palatine tonsil that manifests as a discrete mass is uncommon and continues to pose diagnostic difficulties. The infection has earned the reputation of “the great masquerader” because it readily simulates malignant neoplasms, mycobacterial disease, deep fungal infections, and other chronic inflammatory processes [2-4].

Patients with tonsillar actinomycosis commonly present with unilateral tonsillar enlargement, dysphagia, odynophagia, or cervical lymphadenopathy. These features, together with radiologic findings, frequently prompt a work-up for suspected head-and-neck cancer [2-7]. Imaging characteristics are often nonspecific; therefore, the diagnosis ultimately depends on histopathologic identification of sulfur granules and filamentous bacterial colonies, which are accentuated by periodic acid-Schiff (PAS) and Grocott methenamine silver (GMS) stains [8].

We describe a case of tonsillar actinomycosis in a 67-year-old man who presented with a sizable unilateral tonsillar mass that was radiologically interpreted as an invasive oropharyngeal malignancy. Histopathologic examination and ancillary studies established the infectious etiology, underscoring the necessity of considering infectious causes in the differential diagnosis of tonsillar masses.

## Case presentation

A 67-year-old male with a medical history of type 2 diabetes mellitus, hypertension, and ischemic heart disease sought medical attention because of a right tonsillar mass that had enlarged over several weeks and was associated with throat discomfort. Given the patient’s advanced age, comorbidities, and unilateral tonsillar swelling, oropharyngeal malignancy was considered the leading clinical concern.

**Figure 1:**
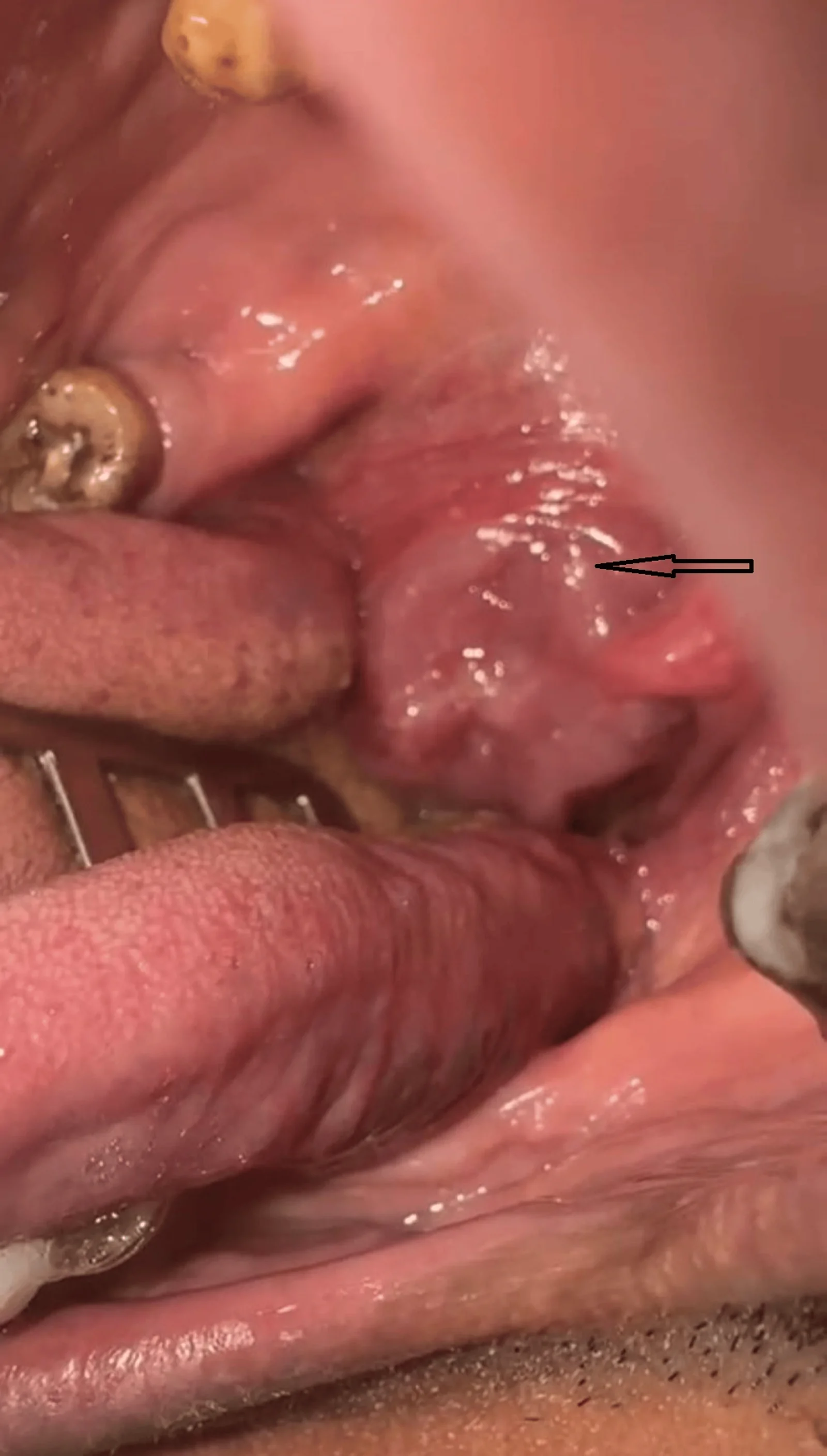
Intraoral endoscopic view of the right tonsillar region showing a mass mimicking an oropharyngeal neoplasm (black arrow).

Clinical photograph taken during an oropharyngeal examination using a flexible endoscope showed a mass in the right tonsillar fossa (Figure 1).

**Figure 2:**
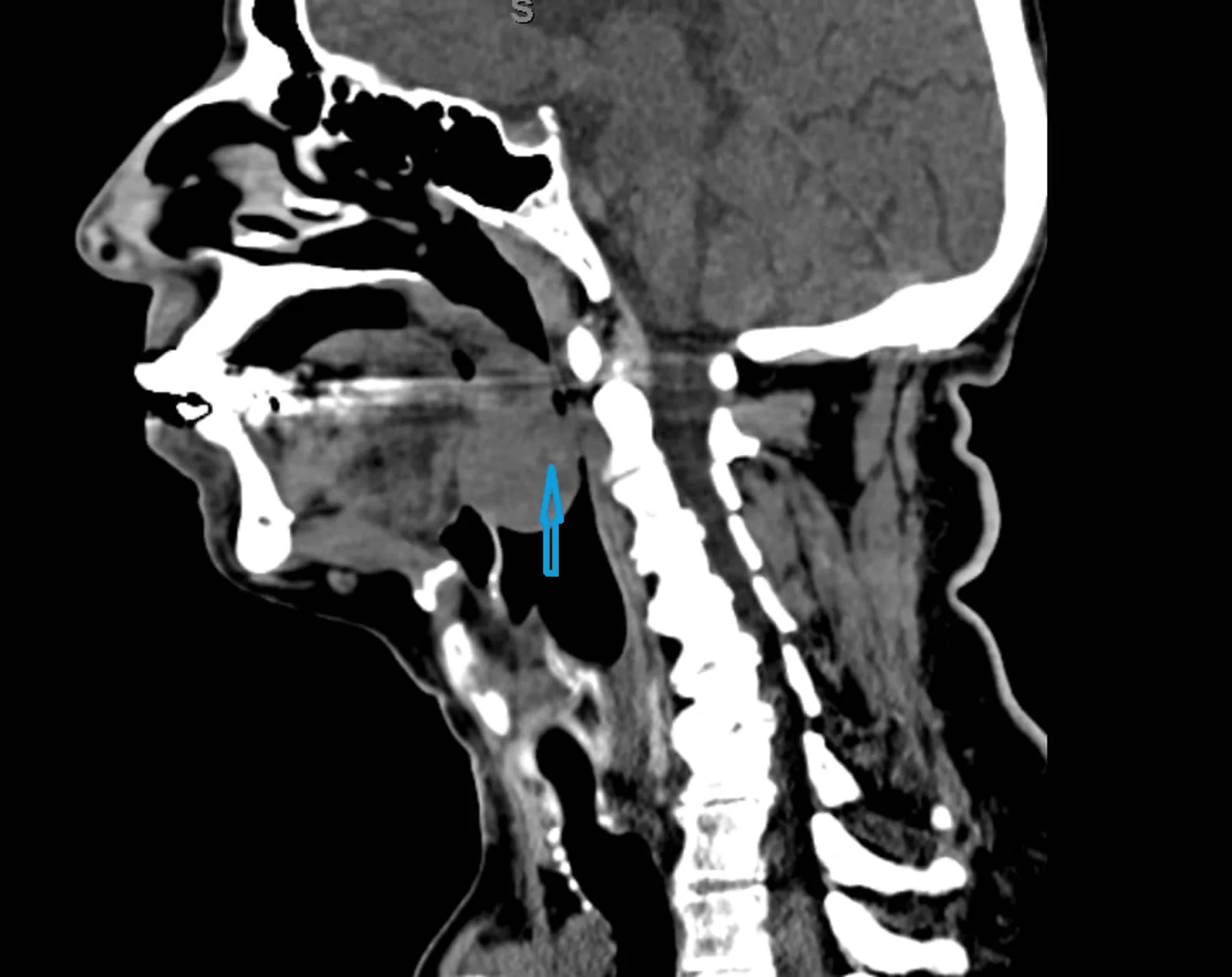
CT image showing homogeneous and intensely enhancing, lobulated, exophytic centered within the right tonsillar fossa (blue arrow). The lesion abutted the right base of the tongue, protruded into the oropharyngeal airway, and extended laterally toward the pterygoid musculature (blue arrow).

CT scan demonstrated a homogeneously and intensely enhancing, lobulated, exophytic mass measuring approximately 42 mm × 24 mm × 47 mm (anteroposterior × transverse × craniocaudal) and centered within the right tonsillar fossa. The lesion abutted the right base of the tongue, protruded into the oropharyngeal airway, and extended laterally toward the pterygoid musculature. These imaging features strongly suggested a primary oropharyngeal neoplasm with local invasion (Figure 2).

The patient underwent surgical excision. Intraoperatively, the mass proved to be well circumscribed without evidence of infiltration into adjacent tissues; the tonsil was removed intact with preservation of its capsule. Postoperative recovery was uneventful after administration of analgesics and antibiotics. Gross examination revealed an enlarged tonsillar specimen measuring 4.5 cm × 3.5 cm × 2 cm with preserved external contours.

**Figure 3:**
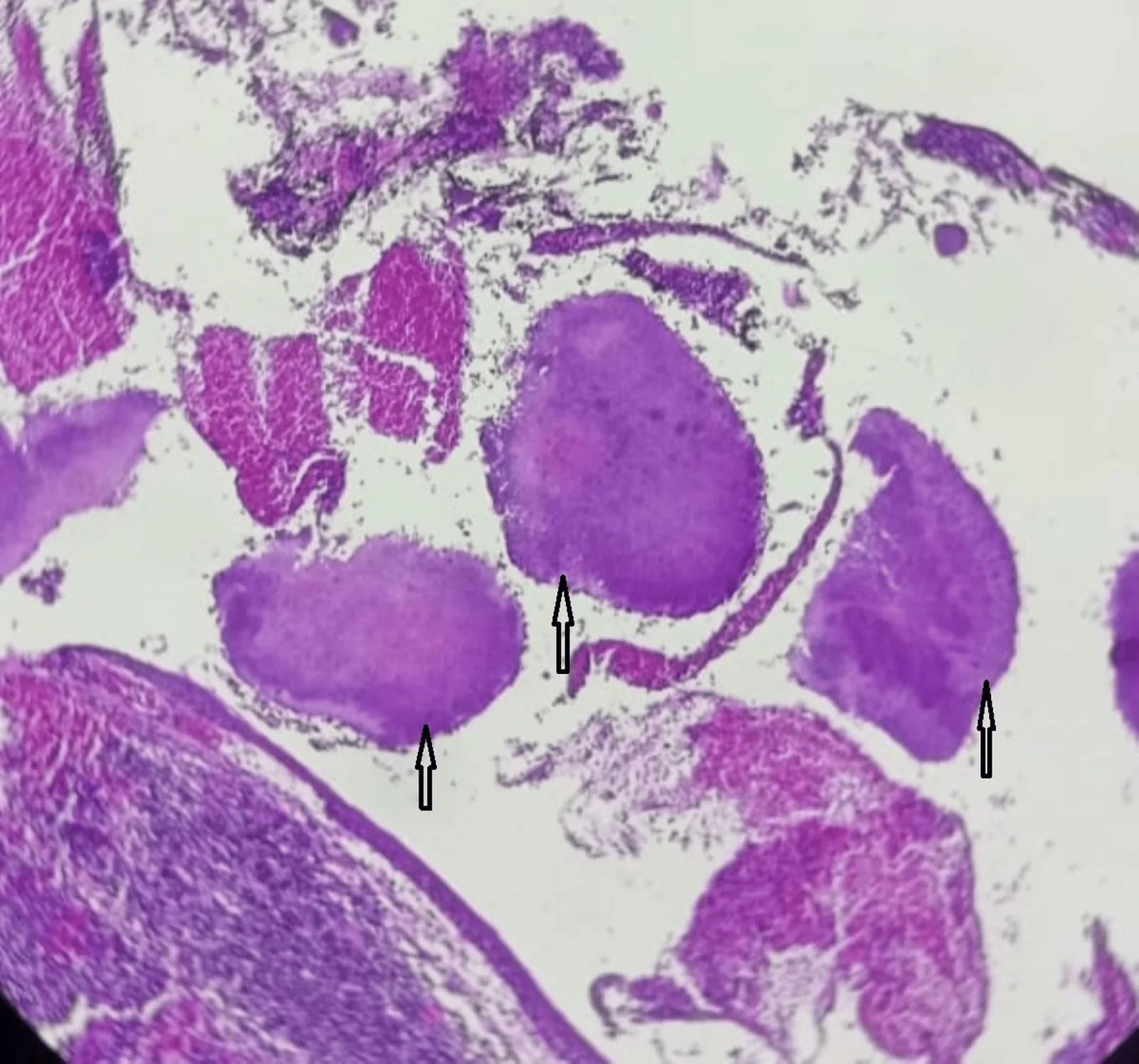
Hematoxylin-and-eosin-stained section shows tonsillar parenchyma containing basophilic radiating, thin, branched and filamentous bacterial colonies (black arrows). x400 magnification.

Microscopic review of hematoxylin-and-eosin-stained sections disclosed tonsillar parenchyma containing basophilic filamentous bacterial colonies surrounded by peripheral eosinophilic club-like material, consistent with sulfur granules. These colonies were accompanied by a mixed acute and chronic inflammatory infiltrate (Figure 3).

**Figure 4:**
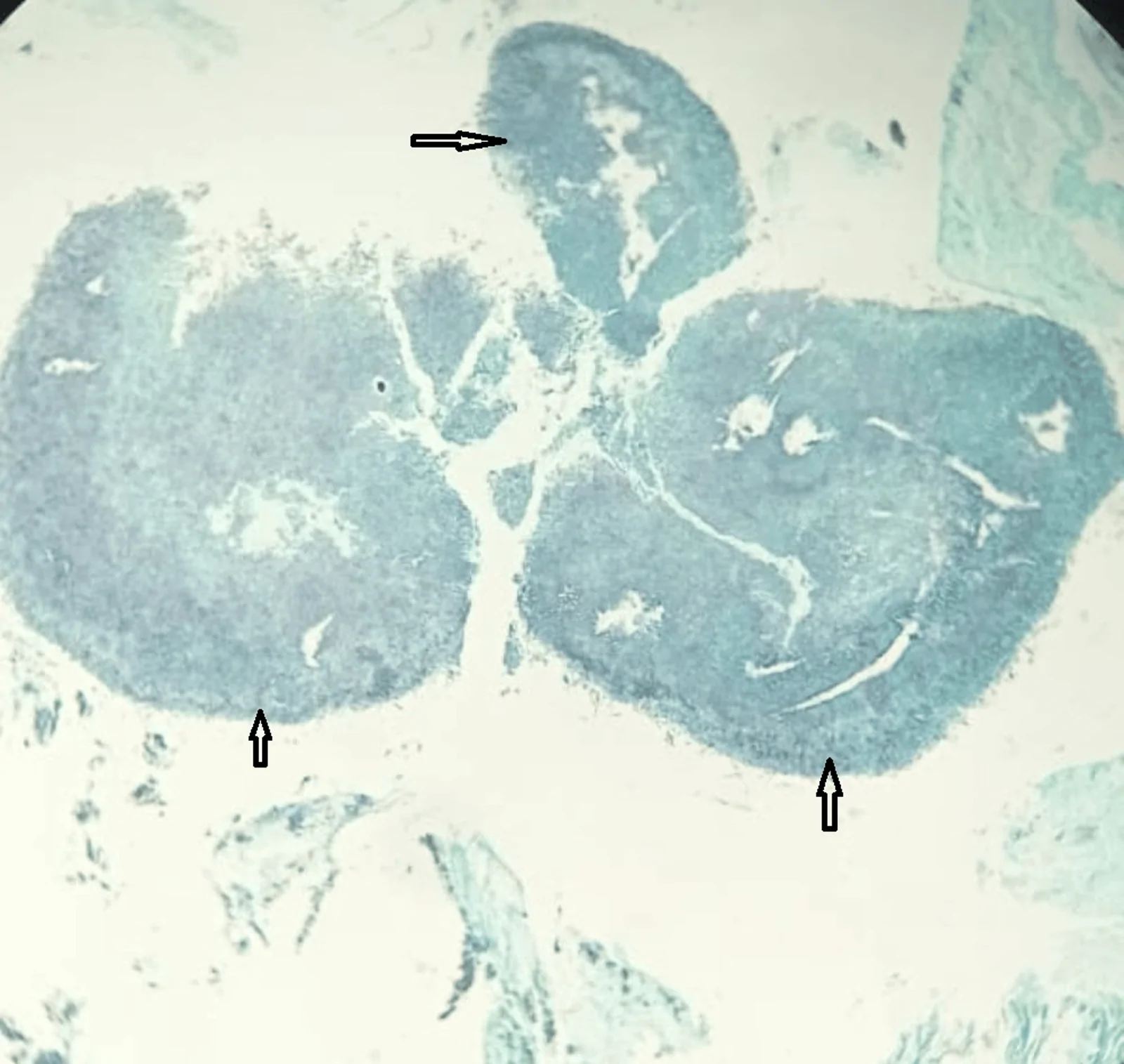
Photomicrograph showing the bacteria colonies (black arrows) stain strongly and diffusely positive for GMS. x400 magnification. GMS: Grocott methenamine silver

**Figure 5:**
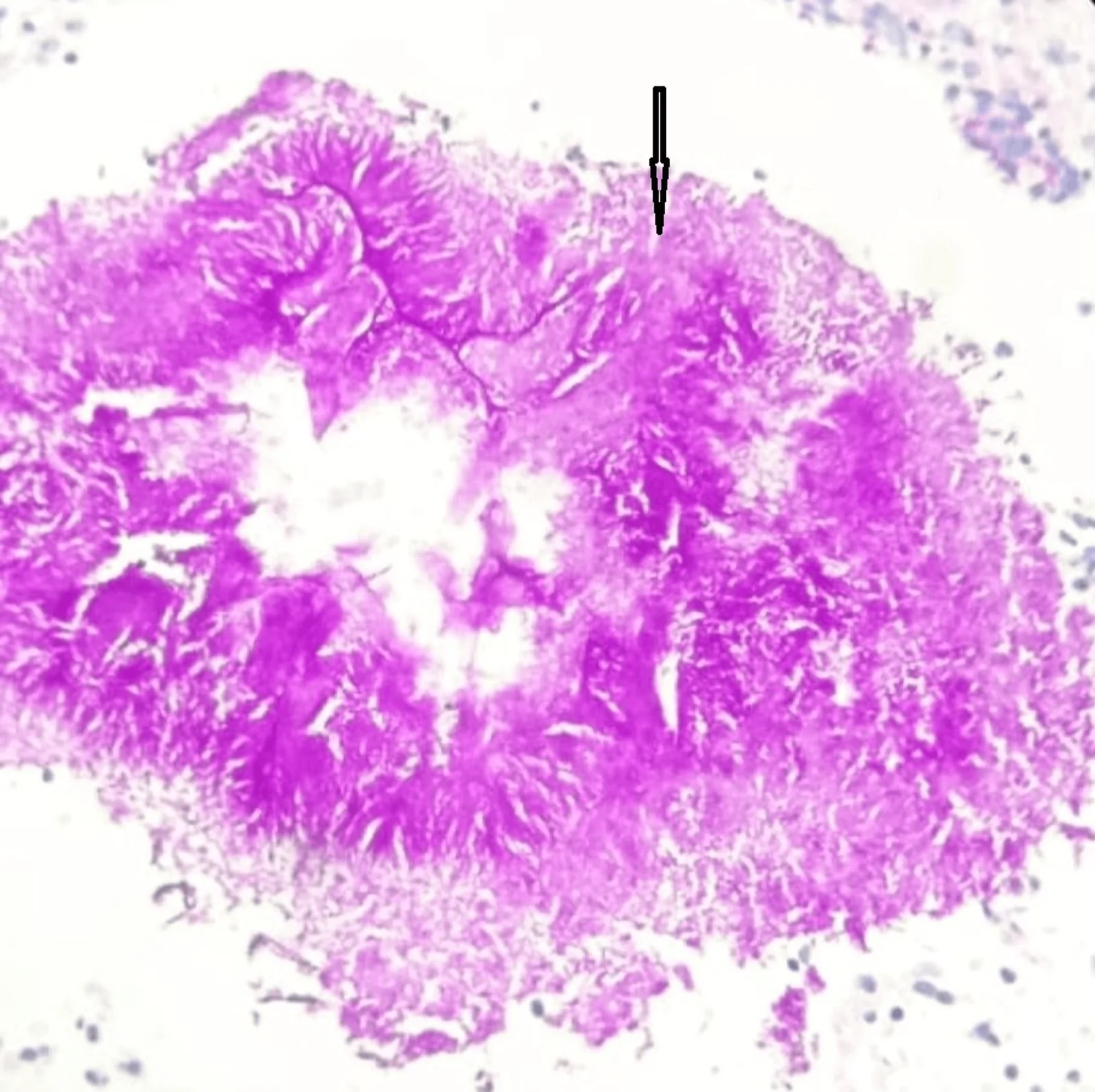
Photomicrograph showing the bacteria colonies (black arrow) stain strongly and diffusely positive for PAS. x400 magnification. PAS: periodic acid-Schiff

PAS and GMS stains highlighted the characteristic bacterial colonies and surrounding amorphic matrix (sulfur granules), confirming actinomycosis (Figures 4, 5).

Because malignancy had been strongly suspected clinically and radiologically, a comprehensive immunohistochemical panel was performed. Staining for cytokeratins, CD3, CD20, CD45, desmin, and p16 yielded negative results, thereby excluding epithelial, lymphoid, myogenic, and HPV-related neoplastic processes. No histologic evidence of squamous cell carcinoma, lymphoma, sarcoma, or other malignancy was identified. On the basis of the characteristic histomorphological features and supportive special stains, a diagnosis of tonsillar actinomycosis was established.

Following surgery and appropriate antibiotic therapy, the patient’s symptoms improved markedly. At subsequent follow-up visits, he remained clinically stable without recurrent symptoms or new lesions.

## Discussion

Actinomycosis is a chronic suppurative granulomatous infection caused chiefly by Actinomyces israelii and related species. These organisms reside as commensals in the oral cavity but become pathogenic once mucosal barriers are breached, permitting deep tissue invasion. Recognized predisposing factors include poor oral hygiene, dental pathology, trauma, immunosuppression, diabetes mellitus, and previous surgery or radiotherapy [1,8,9].

The present case is notable because the lesion closely recapitulated an oropharyngeal malignancy both clinically and on imaging. The patient belonged to the demographic group most commonly affected by head-and-neck squamous cell carcinoma and possessed comorbid conditions that heightened clinical concern. CT scan showed a large, enhancing tonsillar mass with apparent extension into neighboring structures, findings that strongly favored neoplasia. Comparable diagnostic challenges have been reported in the literature for tonsillar, pharyngeal, hypopharyngeal, and laryngeal actinomycosis [2-6,10].

Multiple authors have emphasized the remarkable capacity of actinomycosis to imitate malignant disease. Mohanty documented a case of faucial tonsillar actinomycosis initially regarded as an oropharyngeal tumor [3]. More recently, Navarro et al. described unilateral tonsillar enlargement initially suspected to represent malignancy that was ultimately proven to be actinomycosis after excision and histologic examination [4]. Similarly, Pezier et al. reported invasive tonsillar actinomycosis that clinically masqueraded as tonsillar carcinoma [11]. These observations reinforce the imperative for histopathologic confirmation before embarking on definitive oncologic therapy.

Histopathologic examination remains the diagnostic gold standard. Classic findings consist of sulfur granules formed by intertwined filamentous bacterial colonies encircled by eosinophilic club-shaped material (Splendore-Hoeppli phenomenon). PAS and GMS stains are valuable for highlighting the organisms and distinguishing them from other pathogens. Although microbiologic culture can provide species identification, successful isolation is frequently hampered by prior antibiotic exposure and the fastidious anaerobic requirements of the organisms [1,8].

The differential diagnosis in this case encompassed squamous cell carcinoma (including HPV-associated oropharyngeal carcinoma), lymphoma, and mesenchymal neoplasms. The broad immunohistochemical work-up proved decisive in excluding these entities. Negative cytokeratin staining argued against epithelial malignancy; absence of aberrant lymphoid marker expression ruled out lymphoma; negative desmin staining eliminated a myogenic tumor; and lack of p16 expression lowered the probability of HPV-driven squamous cell carcinoma.

An additional noteworthy finding was the discordance between radiologic and intraoperative appearances. Despite CT scan features suggestive of local invasion, surgery revealed a well-demarcated lesion that was easily excised with an intact capsule and without infiltration of surrounding tissues. This discrepancy illustrates how inflammatory processes can radiologically mimic invasive neoplasms because of surrounding edema and reactive stromal changes.

Standard management of cervicofacial actinomycosis combines surgical excision or debridement with prolonged high-dose penicillin-based antibiotic regimens. In localized tonsillar disease, complete surgical excision can serve both diagnostic and therapeutic purposes, especially when malignancy cannot be excluded preoperatively [2,4,8]. The favorable postoperative course observed in our patient aligns with prior reports indicating that localized tonsillar actinomycosis carries an excellent prognosis when managed appropriately.

Learning points

Tonsillar actinomycosis may manifest as an exophytic oropharyngeal mass with radiologic features suggestive of malignancy, even in elderly patients burdened by diabetes mellitus, hypertension, and cardiac disease. Histopathologic evaluation incorporating special stains (PAS and GMS) together with immunohistochemistry is indispensable for definitive diagnosis and exclusion of neoplasia; microbiologic culture is frequently unrewarding.

Surgical excision combined with prolonged high-dose penicillin therapy constitutes the cornerstone of management, achieving cure and minimizing the risk of recurrence. Actinomycosis should be retained in the differential diagnosis of unilateral tonsillar or oropharyngeal masses to prevent unnecessary radical surgical procedures.

## Conclusions

In summary, tonsillar actinomycosis constitutes a rare yet clinically important benign mimic of oropharyngeal malignancy. Elderly individuals presenting with unilateral tonsillar enlargement and radiologically suspicious masses may undergo extensive oncologic investigations.

Recognition of the distinctive histopathologic features, supported by special stains and exclusion of neoplastic processes through immunohistochemistry, is essential for correct diagnosis. This case underscores the pivotal role of pathologic examination in preventing misdiagnosis and avoiding unwarranted aggressive oncologic intervention.
